# Health Risk Assessment on Hazardous Ingredients in Household Deodorizing Products

**DOI:** 10.3390/ijerph15040744

**Published:** 2018-04-13

**Authors:** Minjin Lee, Joo-Hyon Kim, Daeyeop Lee, Jaewoo Kim, Hyunwoo Lim, Jungkwan Seo, Young-Kwon Park

**Affiliations:** 1 School of Environmental Engineering, University of Seoul, Seoulsiripdaero 163, Dongdaemun-gu, Seoul 02504, Korea; mj_lee@kr.kotiti-global.com; 2 Consumer Product & Environment Business Division, KOTITI Testing & Research Institute, 111 Sagimakgol-ro, Jungwon-gu, Seongnam-si, Gyeonggi-do 13202, Korea; jw_kim@kr.kotiti-global.com; 3 Division of Risk Assessment, National Institute of Environmental Research, Hwangyeong-ro 42, Seo-gu, Incheon 22689, Korea; jhkim0318@korea.kr (J.-H.K.); daeyub22@korea.kr (D.L.); yaho365@korea.kr (H.L.); jkseo2001@korea.kr (J.S.)

**Keywords:** deodorizing product, ingredient chemicals, toxicological endpoint, BHT, human health risk assessment

## Abstract

The inhalation of a water aerosol from a humidifier containing disinfectants has led to serious lung injuries in Korea. To promote the safe use of products, the Korean government enacted regulations on the chemicals in various consumer products that could have adverse health effects. Given the concern over the potential health risks associated with the hazardous ingredients in deodorizing consumer products, 17 ingredients were analyzed and assessed according to their health risk on 3 groups by the application type in 47 deodorizing products. The risk assessment study followed a stepwise procedure (e.g., collecting toxicological information, hazard identification/exposure assessment, and screening and detailed assessment for inhalation and dermal routes). The worst-case scenario and maximum concentration determined by the product purpose and application type were used as the screening assessment. In a detailed assessment, the 75th exposure factor values were used to estimate the assumed reasonable exposure to ingredients. The exposed concentrations of seven ingredients were calculated. Due to limitation of toxicity information, butylated hydroxyl toluene for a consumer’s exposure via the dermal route only was conducted for a detailed assessment. This study showed that the assessed ingredients have no health risks at their maximum concentrations in deodorizing products. This approach can be used to establish guidelines for ingredients that may pose inhalation and dermal hazards.

## 1. Introduction

Personal care products are used widely and regularly by people, often on a daily basis. People use consumer products for household cleaning and personal care because they improve their living and sanitary conditions. In recent years, various studies pointed out that some chemicals found in personal care products, e.g., phthalate, heavy metals (e.g., zinc, lead, and arsenic), methanol, hydroquinone, and 1,4-dioxane, may be associated with a risk of allergies, endocrine disruption, neurotoxicity, birth defects, or cancer [1,2,3,4,5].The major route for consumer exposure to the vast majority of household products is through inhalation and dermal contact. In addition to skin contact, spray products require considerations with regard to potential inhalation for building a robust and reliable safety assessment [6]. Ingredient chemicals, such as fragrances and biocides, may have adverse health effects (e.g., humidifiers, including polyhexamethylene guanidine chloride (PHMG), caused serious lung injuries and deaths). In 2013, the Korean government adopted regulations on chemicals in a range of consumer products that might have adverse health effects to promote the safe use of these products [7]. A consumer product (substance, mixture or article) is a product that can be purchased from retail outlets by the public. The manufacturer and importer of the substances, being part of do-it-yourself products sold by retailers, should also determine that consumer use has been assessed and safe consumer use can be assured. Internal exposure to chemicals in consumer products has been suspected to cause cancer [8], skin rashes, allergies [9,10,11], eye irritation, respiratory irritation [6,12,13,14,15], and allergic dermatitis [4,8,16,17].

This study presents an approach to compile common principles for an exposure assessment and risk assessment for consumer products, such as deodorizing products. A consumer exposure assessment was carried out according to the guidance from the information requirement and chemical safety assessment [18], which was described as an efficient, step-wise, and iterative procedure (e.g., characterize the substance, determine the scope of exposure assessment, build/retrieve the contributing use scenario, estimate the event exposure, and carry out risk characterization). The National Institute of Environment Research (NIER) has established the guidelines for human health risk assessments of consumer products, including exposure factors and exposure equations, to estimate the potential human risk of the ingredients used in consumer products [19].

Butylated hydroxyl toluene is used widely as an antioxidant to preserve and stabilize the freshness, nutritional value, flavor, and color of foods and animal feed products [20]. Siloxanes are used widely in consumer products, such as paints, cosmetics, and household products, as well as in medical products. Recently, however, various studies have pointed out that some siloxanes may have endocrine disrupting properties and effects on reproduction, which may cause concern regarding their effects on humans and the environment [21,22,23]. The first step related to a hazard assessment is to collect and generate information on the intrinsic properties of the ingredients and determine the toxicological endpoints, including taking the use pattern and routes of exposure into account. The endpoints also consider the physicochemical properties of ingredients [18]. This study identified the concentration of hazardous ingredients in deodorizing products and evaluated the risk characterization for dermal and inhalation exposure, based on “the worst-case scenario” and related to a single consumer product use. The target ingredients were evaluated for a human health risk assessment following the single use of a selected consumer product.

## 2. Materials and Methods

### 2.1. Preparation of the Target Products and Ingredients

To identify how the ingredients were used in the various products, 47 deodorizing products (used in indoor air and vehicle interiors, for fabrics and shoes, and for air conditioners and other purpose) were purchased online and from a supermarket based on the results of a survey conducted to elucidate the products commonly used on the Korean market [24]. These deodorizing products comprised 31 spray type products (aerosol and trigger sprays) and 16 other application types (liquid, and fumigation) (Table 1). The Korean Ministry of Environment (KME) has established safe guidelines for risk-concerning products for residential consumer use, and 13 substances for deodorizing products are regulated already as the safe guideline. In this study, hazardous chemicals used as ingredients in deodorizing products except for regulated substances were selected and identified. A list of hazardous ingredients was surveyed from the manufacturing companies of these deodorizing products in Korea by the KME.

### 2.2. Concentration Determination of the Target Ingredients

The purchased deodorizing products samples were prepared, extracted, and analyzed according to the standard operation of the analytical procedure (SOP) developed by the National Institute of Environment Research [25]. A total of 17 substances were analyzed in deodorizing products; Table 2 shows target chemicals, chemical information, pre-treatment method of products, and analysis instrument for target chemical analysis in deodorizing products.

#### 2.2.1. Chemicals

The solvents and reagents were of analytical reagent grade. Butylated hydroxytoluene (BHT, 99.90% purity), benzaldehyde (99.54% purity), octamethylcyclotetrasiloxane (D4, 97.00% purity), and isopropyl alcohol (IPA, 100% purity) were purchased from Sigma-Aldrich (St.Louis, MI, USA). 1,4-dichlorobenzene (99.50% purity) and methanol (99.90% purity) were obtained from Dr. Ehrenstorfer. Dimethyl phthalate (DMP, 99.50% purity), diethyl phthalate (DEP, 99.50% purity), diisobutyl phthalate (DIBP, 99.50% purity), Di(-n-)butyl phthalate (DBP, 99.50% purity), butyl benzyl phthalate (BBP, 98.60% purity), bis(2-ethyl hexyl) phthalate (DEHP, 99.50% purity), di-n-octyl phthalate (DNOP, 99.50% purity), diisononyl phthalate (DINP, 100.00% purity), diisodecyl phthalate (DIDP, 99.50% purity), and naphthalene (99.50% purity) were provided by Chemservice (Herrnsheim Hauptstr, Germany). Zinc was purchased from Accustandard (New Haven, CT, USA).

#### 2.2.2. Equipment

The headspace (HS) used was a TurboMatrix 40 Trap (Perkin Elmer, ‎Waltham, MA, USA). Gas chromatography (GC) was carried out using an Agilent 6890 gas chromatograph (Santa Clara, CA, USA) with flame ionization detection (FID) (Shimadzu, Kyoto, Japan) and mass spectrometry (MS, Shimadzu MSD QP-2010 Ultra mass spectrometer, Kyoto, Japan) detection. High performance liquid chromatography (HPLC) was carried out using an Ultimate 3000 liquid chromatograph (LC, Thermo Fisher, Waltham, MA, USA). Inductively coupled plasma-MS (ICP-MS) analysis was carried out using an iCAP Q (Thermo Fisher, Waltham, MA, USA).

#### 2.2.3. Analysis of Ingredients

A market survey was conducted to elucidate the products that are commonly used in the Korean market. Based on the results of the survey, 47 products were purchased and divided into 9 groups by product usage, and 5 groups by application type. To select target ingredients, information on ingredients used in deodorants was obtained from the manufacturing companies. Then, 17 ingredients were selected by the mixed proportioning of surveyed products, frequency of use, and market share of the products. The solvents and reagents were of analytical grade. Butylated hydroxyl toluene and octamethylcyclotetrasiloxane were analyzed simultaneously by GC/MS after sonication extraction with n-hexane [26,27]. Naphthalene and phthalates group were quantified using GC-MS. 1,4-Diclorobenzene and isopropyl alcohol were quantified simultaneously using headspace GC-MS [28]. Methanol was analyzed by GC-FID, and zinc was analyzed using ICP-MS. Benzaldehyde was derived with 2,4-dinitrophenylhydrazine (DNPH) and analyzed by HPLC [29]. In all cases of analysis, the standard operation of procedure (SOP) developed by KME was followed and adherence to quality assurance/quality control requirements was maintained, including method blank, reagent blank, instrument detection limit, and calibration curve. The summary of analytical methods that were used is presented in Table 2.

### 2.3. Quality Assurance/Quality Control and Recovery Study

All analytical procedures including recovery study were monitored using the QA/QC guidelines in the Korean official method on the National Institute of Environment Research (KNLIC) [25]. The limits of detection (LOD) and quantification (LOQ) of each analysis were calculated as the analysis concentration corresponding to three and ten times, respectively, the standard deviation of ten independent measurements of blank or low concentrations. To verify to accuracy and precision of the analytical procedure, the recovery studies were carried out. The recovery of target ingredients added to samples without the target chemicals was carried out. Products samples were analyzed before and after addition of 100 and 200 mg of target ingredients to 100 g of the products samples. According to guidelines, quality control target value of precision was a relative standard deviation smaller than 30%. Therefore, goal value of accuracy was 70–130%.

### 2.4. Toxicity Information and Dose-Response for the Target Ingredients

The target routes of exposure were considered to be inhalation and dermal route according to usage purpose and application types of deodorizing products. An evaluation of the toxicological data was carried out in relation to the respiratory and irritant effects of short- and long-term exposure to the ingredients under investigation. The official toxicological reports and studies (e.g., EU ECHA Dossier, and OECD SIDS report) were collected for each ingredient. In the next step, the toxicity reference values (e.g., chronic no observed adverse effect concentration (NOAEC) and no observed adverse effect (NOAEL)) were calculated according to the official guidance [30]. The uncertainties in the extrapolation of the experimental data to a real human exposure situation, inter-species and intra-species differences, differences in the duration of exposure, and differences in the toxicological value (e.g., LOAEL and NOAEL) were considered [30]. Table 3 summarizes the toxicity, assessment factor, reference value, and target margin of exposure (MOE). The target MOEs for screening and detailed risk assessments were determined according to the risk assessment guideline for consumer products developed by the National Institute of Environmental Research [19]. According to the guidelines, the target MOE was established for each ingredient by intra- and inter-species analysis, together with other factors. The ingredient may pose a health risk if the MOE of the ingredient is lower than the target MOE [18].

#### Butylated Hydroxyl Toluene

The NOAEL value of butylated hydroxyltoluene for oral exposure is 25 mg/kg/day. The original toxicological value was adjusted directly and divided by 1 (from chronic to chronic). According to the European Center for Ecotoxicology and Toxicology of Chemicals TR No. 86 [31], a route to route extrapolation is only feasible for systemic analysis, and not for local effects. In addition, the dose rate and toxicokinetic information should be considered. In this study, only the route-to-route extrapolation was applied to the oral to dermal route, without default values due to the limited dermal toxicological information [24].

### 2.5. Hazard Identification and Exposure Assessment for Target Ingredients

This study considered the exposure to products through two pathways: inhalation and dermal contact. The procedure for a risk assessment in this study followed a previous study [24]. Exposure to each product was estimated using equations based on a model developed by the National Institute of Environment Research [25]. The risk assessment models consisted of two stages. To assess the health risk from the exposure level, the MOE and hazard quotient (HQ) were calculated for the ingredients. The MOE is defined as the ratio of the NOAEL for the critical effect to the theoretical, predicted or estimated dose or concentration [32]. HQ is defined as a reference dose. The MOE is defined as a reference point derived from the dose-response relationship, divided by the estimated human exposure level [33]. In this study, the MOE was calculated from the ratio of the chronic NOAEL to the human exposure level. When toxicological values (e.g., LOAEC, LOAEL, and acute and sub-chronic values) were adjusted to chronic NOAEC and NOAEL, exposure duration, frequency of treatment, and exposure were considered [18]. In addition, the target MOE was established for each ingredient by intra- and inter-species analysis, together with other factors. If the MOE of the ingredient is lower than the target MOE, the ingredient may pose a health risk [18]. Because the purpose of Tier 1 assessment was screening of the risk of the substance, the MOE and HQ were set to 10 times higher than the normal MOE. If the ingredient in the product posed a risk, the ingredient was selected and subjected to Tier 2 assessment. If ingredients did not have toxicological information or were not detected, exposure assessment was not performed. Tier 1 assessment is usually used to screen consumer exposure based on the summation of high percentile product consumptions, amounts per use, and concentration of ingredients in products to assume a worst-case exposure scenario [34]. To determine the inhalation and dermal exposed dose of the target ingredients, the 95th exposure factor values in the Korean consumer exposure factors were inserted into the calculation equation according to the exposure route (e.g., frequency of use, duration of use, and amount used per application) [19] (Table 4). For the inhalation and dermal exposure assessments, the exposure dose through the inhalation and dermal routes were calculated using the equations in Table 5. Abs (absorption ratio to body) was assumed to be 100%. V (volume of space, m^3^) was assumed to be 33.3 m^3^, which is the mean size of a living room in Korea, as reported by the Korean consumer exposure factors. BW (body weight) was assumed to be 64.2 kg, which is the mean weight of a Korean adult, as reported by the Korean exposure factors handbook [35]. A detailed assessment estimates the general consumer exposure based on the summation of a reasonable percentile product consumption, i.e., amount per use, concentration of ingredients in the products, ventilation rate, and product characteristics (e.g., airborne fraction). Some chemicals did not show enough toxicity information and endpoint for inhalation or dermal exposure Tier 2 assessment. Analysis result of some chemicals showed very low detection rate in products. Therefore, we determined a suitable target chemical, BHT, for further Tier 2 assessment. The 75th exposure factor values in the Korean consumer exposure factors were used in the model involving the exposure routes (Table 4). The National Institute of Environment Research [25] also developed the scenario for the exposure through the inhalation and dermal routes by the products. A previous study specified the other factors associated with Korean consumer exposure, i.e., volume of living spaces, ventilation rate of living spaces, and absorption amount to skin [23].

## 3. Results

### 3.1. Analysis of Hazardous Ingredients in Deodorizing Products

Table 6 lists the correlation coefficient, regressive equation, linear range, and LOQ for the 17 target ingredients. In a previous study, the respiratory and irritative health effects of fragrance chemicals and biocides in deodorizing products were assessed. The present study considered the hazardous ingredients in deodorizing products. In total, 17 hazardous ingredients were analyzed in 47 deodorizing products of spray, trigger, liquid, and fumigation application types. Seven ingredients in the products were analyzed among the 17 ingredients in the deodorizing products in this study. Table 7 lists the number of analyzed products and their range of ingredient concentrations. The detection rate of hazardous ingredients ranged from 0.13% to 63.83% in mostly the trigger application type products. The detection frequency of butylated hydroxytoluene was relatively high, i.e., it was found in 30 out of the 47 products. The detection rates of the ingredients were in the following order: butylated hydroxytoluene 63.83%, octamethyl cyclotetrasiloxane 8.51%, zinc oxide and di-n-octyl phthalate 6.38%, and isopropyl alcohol 4.26%. Isopropyl alcohol in one deodorizing product showed maximum concentrations greater than 23% (236,266 mg/kg). Only one deodorizing product each contained dibutyl phthalate and bis(2-ethyl hexyl) phthalate at 358.86 mg/kg and 215.95 mg/kg, respectively (Table 7). The other ingredients were detected as below the limit of quantitation.

### 3.2. Determination of Toxicological Endpoint

Consumer exposure for deodorizing products considered two exposure routes, inhalation and dermal; each exposure route was calculated separately. The target exposure routes in this study were also inhalation and dermal. In the case of an absence of toxicological information on the target chemicals or undetectable compounds were not studied. The toxicological data were assessed based on long-term exposure to the ingredients and exposure routes. The ECHA registration dossier of ingredients was used to evaluate the toxicity of zinc oxide, isopropyl alcohol, and bis(2-ethyl hexyl)phthalate. The toxicity of butylated hydroxyl toluene and the oral toxicity data of isopropyl alcohol were derived from the OECD-generating profile (the screening information dataset (SIDS) initial assessment profile). To determine the reference toxicity values for inhalation, the uncertainty factor was adjusted to 2 (sub-chronic toxicity value to long-term inhalation exposure) or 6 (sub-acute toxicity value to long-term inhalation exposure) and the NOAEL or the NOAEC from the results of short-term inhalation exposure in rats. The inter-species extrapolation for the inhalation route was 2.5, and an intra-species variation of 10 was used to determine the target MOE. The target MOE using the oral NOAEL in rats with the inter-species extrapolation of 10, was calculated to be 100. The reference values determined for the inhalation of zinc oxide, isopropyl alcohol, and bis(2-ethyl hexyl)phthalate were 0.1 mg/m^3^, 119.8 mg/m^3^, and 2.1 mg/m^3^, respectively. To determine the reference toxicity for dermal exposure, the inter-species extrapolation for the dermal route was 6. Table 3 provides a brief overview of toxicity studies used to determine the endpoint in the risk assessments. The determined reference values for the dermal route of butylated hydroxyl toluene, zinc oxide, isopropyl alcohol, and bis(2-ethyl hexyl)phthalate were 25 mg/kg/day, 0.4 mg/kg/day, 240 mg/kg/day, and 28.9 mg/kg/day, respectively. The target MOE for a detailed risk assessment adopted was 10 times higher than the target MOE for a screening risk assessment (Table 3).

### 3.3. Calculation of Exposure

To assess the inhalation and dermal exposure for screening, the exposure concentration by the target ingredients in the spray and trigger type deodorizing products were calculated using the equations in Table 8 with a worst-case scenario and the 95th exposure factor values in Korean consumer exposure factors. According to the assessment result through the dermal route, butylated hydroxyl toluene may pose a health risk. The MOE for butylated hydroxyl toluene was lower than the target MOE. None of the other ingredients have toxicological information and the detection rate in the deodorizing products was too low; consequently, exposure assessment could not be performed.

### 3.4. Exposure Assessment of the Dermal Route for Butylated Hydroxyl Toluene

Table 8 lists the detailed dermal assessment by butylated hydroxyl toluene. Different exposure models were used according to the product purpose. Butylated hydroxyl toluene was determined for the spray and trigger used for fabrics and shoes, and the MOEs of this ingredient were found to be much higher than the target MOE because of the low exposure dose and relatively high reference toxicity. The target ingredients studied in the selected types of deodorizing products were considered to be adequately safe, because the calculated MOE of the ingredient was greater than the target MOE.

## 4. Discussion

A previous study reported human health risk assessment studies conducted for consumer exposure to the ingredients, biocidal and fragrance chemicals, used in deodorizing products according to Tier 1 (screening) and Tier 2 (detail) assessment processing [24]. In this study, exposure assessment studies were conducted for consumer exposure (non-professional users) to the ingredients used in deodorizing products, including hazardous chemicals, and an approach to compile the common principles for an exposure assessment and risk assessment for a consumer products evaluation was presented. The aim of this study was to make a reasonable suggestion for standards for the chemicals used in consumer products through a risk assessment study. This comprehensive study provides ways for the mandatory regulation of consumer products focused on humans, e.g., a risk assessment for hazardous ingredients in deodorizing products being marketed.

Risk assessment studies of consumer products have recently become a hot issue in Korea. Because of unidentified fatal lung disease caused by the chemical disinfectants used in household humidifiers [36], the Korean Ministry of Environment and the National Institute of Environmental Research conducted human risk assessment studies to assess hazardous ingredients, most of which are used in consumer products. The Korean Ministry of Environment established safe guidelines for consumer products and is regulating ingredients in consumer products strongly [19]. For a risk assessment study, the assessment procedure of a previous study was followed (e.g., collecting toxicological information, hazard identification, and Tier 1 and Tier 2 assessments for inhalation and dermal routes). According to the survey results involving deodorizing products manufacturing companies, this study selected and analyzed 17 hazardous ingredients included in the deodorizing products. In the case of deodorizing products, inhalation and dermal exposure are considered to be the main exposure routes to consumers [25,37]. Quantitative considerations in risk assessments include dose–response assessments, exposure assessments, and characterization of uncertainty. Reliable exposure factors are essential to determine the health risks posed by the ingredients in deodorizing products [38,39].

Butylated hydroxyl toluene is a widely used anti-oxidant additive and an ingredient studied for its potential toxicity. Butylated hydroxyl toluene can improve the stability of consumer products [40] and is permitted as a direct or indirect food additive [41]. Although a variety experimental studies have been reported for several ingredients used in consumer products, e.g., butylated hydroxyl toluene, there are no data for humans. The limited toxicity data for long-term studies may impede a human risk assessment study for target ingredients. The absence of sufficient chemical specific inhalation toxicity data is one of the limitations of a risk assessment study for inhalation. In some cases of a risk assessment for dermal exposure, only the route to route extrapolation was applied for the oral to dermal route without default values due to the limited dermal toxicological information. The hazardous ingredients studied were considered in selected types of deodorizing products, and were found to be adequately safe after a consumer risk assessment because the calculated MOEs of the ingredients were greater than the target MOEs.

## 5. Conclusions

This study analyzed the hazardous ingredients contained in household spray and trigger deodorizing agents and human health exposure assessments. Long-term respiratory exposure to the ingredients may lead to enhanced health risks, such as respiratory problems. Considering the health hazard of household spray and trigger products, proper regulations should be developed to reduce the potential risks associated with the use of those products. Moreover, effort is needed to routinely monitor the consumer’s health risk stemming from long-term use. Because of the difficulties associated with the definition of uses and a complete risk assessment on the government level for most existing and new products, a concerted management effort is needed from both regulatory agencies and manufacturers of chemicals and chemical-containing products. Health risk assessments using exposure scenarios are essential to reflect the current use of chemicals in relevant countries, the detailed information of which can be provided mainly by manufacturers and downstream user groups. In the present study, the respiratory and dermal effects were assessed for the ingredients that consumers were exposed to during the consumer use of deodorizing products. In the “most representative worst-case scenario”, the exposure scenarios used reflected the worst cases for the deodorizing products evaluated. The risk assessment approach discussed in this study should be used to establish improved guidelines for specific ingredients in consumer products, and for setting limits for newly developed raw materials that might pose dermal and inhalation hazards.

## Figures and Tables

**Table 1:** List of deodorizing agents studied.

Sample ID	Product Usage	Application Type	No. of Products
D-S-I-1~7	For indoor air & vehicle interior	Spray (aerosol and trigger spray)	7
D-S-A-1~11	For fabric and shoes		11
D-S-C-1~7	For air conditioner		7
D-S-W-1~2	For food waste		2
D-S-S-1~4	For sick house syndrome		4
D-L-I-1~5	For indoor air & vehicle interior	Liquid	5
D-L-A-1~6	For fabric and shoes		6
D-L-T-1~2	For toilet and car air conditioner		2
D-F-I-1~3	For indoor air & anti-bacteria	Fumigation	3
total			47

**Table 2:** Information on 17 target hazardous ingredients investigated in this study.

Order	Chemicals	Formula	MW (g/mol)	CAS. No.	Pre-Treatment Method	Analysis Instrument
1	Butylated hydroxyl toluene(BHT)	C_15_H_24_O	220.35	128-37-0	Sonication	GC-MS
2	Octamethylcyclotetrasiloxane (D_4_)	C_8_H_24_O_4_Si_4_	296.616	556-67-2	Sonication	GC-MS
3	Naphthalene	C_10_H_8_	128.1705	91-20-3	Sonication	GC-MS
4	Dimethyl phthalate (DMP)	C_10_H_10_O_4_	194.184	131-11-3	Sonication	GC-MS
5	Diethyl phthalate (DEP)	C_12_H_14_O_4_	222.24	84-66-2	Sonication	GC-MS
6	Diisobutyl phthalate (DIBP)	C_16_H_22_O_4_	278.35	84-69-5	Sonication	GC-MS
7	Dibutyl phthalate (DBP)	C_16_H_22_O_4_	278.35	84-74-2	Sonication	GC-MS
8	Benzyl butyl phthalate (BBP)	C_19_H_20_O_4_	312.37	85-68-7	Sonication	GC-MS
9	Bis(2-ethyl hexyl) phthalate (DEHP)	C_24_H_38_O_4_	390.56	117-81-7	Sonication	GC-MS
10	Di-n-octyl phthalate (DNOP)	C_24_H_38_O_4_	390.56	117-84-0	Sonication	GC-MS
11	Diisononyl phthalate (DINP)	C_26_H_42_O_4_	418.609	68515-48-0	Sonication	GC-MS
12	Diisodecyl phthalate (DIDP)	C_28_H_46_O_4_	446.67	68515-49-1	Sonication	GC-MS
13	Isopropyl alcohol(IPA)	C_3_H_8_O	60.1	67-63-0	-	HS-GC-MS
14	1,4-dichlorobenzene	C_6_H_4_C_l2_	146.998	106-46-7	-	HS-GC-MS
15	Methanol	CH_3_OH	32.04	67-56-1	Sonication	GC-FID
16	Benzaldehyde	C_7_H_6_O	106.121	100-52-7	Derivatization	HPLC
17	Zinc oxide (analyzed as zinc)	ZnO	81.408	1314-13-2	Microwave	ICP-OES

**Table 3:** Summary of toxicological end-point and default chronic NOAEL for the ingredients studied.

Chemicals	Referenced Value (Chronic NOAEL)	Toxicity Value	Assessment Factors	Target Exposure Route (Target MOE)
Butylated hydroxyl toluene	25 mg/kg/day (oral to dermal)	NOAEL = 25 mg/kg/day (141–144 weeks/rat, Oral) ^b^	Chronic to Chronic:1	Dermal (sc: 1000, de: 100)
Zinc oxide	0.1 mg/m^3^ (inhalation)	NOAEL = 1.5 mg/m^3^ (3 months/rat, inhalation) ^a^	Sub-chronic to Chronic:2 Intra-species:10 Inter-species:2.5	Inhalation (sc: 250, de: 25)
	0.4 mg/kg/day (dermal)	NOAEL = 75 mg/kg/day (28 days/rat, dermal) ^a^	Sub-acute to Chronic:6 LOAEL to NOAEL:3 Intra-species:10 Inter-species:10	Dermal (sc: 1000, de: 100)
Isopropyl alcohol	119.8 mg/m^3^ (inhalation)	NOAEL = 1342 mg/m^3^ (13 weeks/rat, inhalation) ^a^	Sub-chronic to Chronic:2 Intra-species:10 Inter-species:2.5	Inhalation (sc: 250, de: 25)
	240 mg/kg/day (oral to dermal)	NOAEL = 240 mg/kg/day (28 days/rat, oral) ^b^	Intra-species:10 Inter-species:6	Dermal (sc: 600, de: 60)
Bis(2-ethyl hexyl)phthalate (DEHP)	2.1 mg/m^3^ (inhalation)	NOAEC = 50 mg/m^3 a^	Sub-acute to Chronic:6 Intra-species:10 Inter-species:2.5	Inhalation (sc: 250, de: 25)
	28.9 mg/kg/day (oral to dermal)	NOAEL = 28.9 mg/kg/day (104 weeks/rat, oral) ^a^	Chronic to Chronic:1 Intra-species:10 Inter-species:10	Dermal (sc: 1000, de: 100)
Other ingredients have no available toxicological information for inhalation and dermal exposure				

NOAEL: no observed adverse effect level; NOAEC: no observed adverse effect concentration; LOAEC: lowest observable adverse effect concentration; MOE: margin of exposure, sc: screening; de: detail. ^a^ European Chemicals Agency (ECHA), registration dossier. ^b^: Organization for Economic Co-operation and Development (OECD), Screening Information Dataset (SIDS) report.

**Table 4:** Exposure scenario parameter of deodorizing agents adjusted for Korean consumer circumstances [25].

Products	Application Types	Exposure Factors	Median Range	S.D.	Percentile			
					5th	50th	75th	95th
For fabric	Trigger	Frequency of use (use/day)	0.45	0.76	0.01	0.17	0.43	2.00
		Duration of use (min/use)	1.29	1.68	0.05	0.50	1.50	5.00
		Duration of spraying (s/use)	2.61	2.26	0.57	1.71	2.85	5.70
		Amount used per application (g/s)	0.83	0.61	0.25	0.54	1.26	1.79
Exposure factors for fabrics are the worst-case factors (products for shoes is applied using factors for fabric)								
For indoor air	Trigger	Frequency of use (use/day)	0.65	1.16	0.01	0.29	1.00	2.15
		Duration of use (min/use)	2.00	2.76	0.08	1.00	2.03	10.04
		Duration of triggering (s/use)	2.85	2.37	0.60	1.80	3.00	7.89
		Amount used per application (g/s)	0.55	0.29	0.18	0.60	0.77	0.95
Exposure factors for indoor air are the worst-case factors (products for vehicle interior, toilet and others are applied using factors for indoor air)								
For air-conditioner	Trigger	Frequency of use (use/year)	4.61	4.99	1.00	2.00	6.00	18.60
		Duration of use (s/use)	262.90	237.25	9.35	180.0	600.0	616.15
		Duration of spraying (s/use)	2.74	1.99	0.58	1.74	3.63	5.80
		Amount used per application (g/s)	1.02	0.05	0.99	0.99	1.03	1.06
Liquid diffuser		Amount of emission (g/h)	0.20	0.23	0.00	0.11	0.28	0.61

**Table 5:** Scenarios for the exposure and calculation of inhalation and dermal exposed dose.

Exposure	Cal Equ.	Chemicals	Application	Exposed Dose
Inhalation exposure	C_a_ = A_p_·W_f_/V C_inh_ = C_a_·Abs·t·n/24	Butylated hydroxyl toluene	Trigger type	0.0071 mg/m^3^
		Zinc oxide		0.0301 mg/m^3^
		Octamethyl cyclotetrasiloxane		0.0101 mg/m^3^
		Isopropyl alcohol		10.981 mg/m^3^
		Dibutyl phthalate		0.0166 mg/m^3^
		Bis(2-ethyl hexyl)phthalate		0.0099 mg/m^3^
		Di-n-octyl phthalate		0.0046 mg/m^3^
Dermal exposure	D_der_ = A_p_·W_f_·Abs·n/BW	Butylated hydroxyl toluene	Trigger type	0.0489 mg/kg/day
		Zinc oxide		0.2062 mg/kg/day
		Octamethyl cyclotetrasiloxane		0.0692 mg/kg/day
		Isopropyl alcohol		75.097 mg/kg/day
		Dibutyl phthalate		0.1137 mg/kg/day
		Bis(2-ethyl hexyl)phthalate		0.0683 mg/kg/day
		Di-n-octyl phthalate		0.0327 mg/kg/day

C_a_: concentration of the substance in the air (mg/m^3^); A_p_: amount of product use (mg); W_f_: fraction of a specific substance in product; C_inh_: exposure concentration via inhalation (mg/m^3^); t: duration of use (h); n: frequency of use; V: volume of space (m^3^); Abs: absorption ratio to body; D_der_: dermal exposure dose for spray products (mg/kg/day); BW: body weight.

**Table 6:** Regression equations, correlation coefficients (R^2^), linear range and limit of quantification (LOQ) of 17 target ingredients studied.

Order	Chemicals	Target Ion (*m/z*)	Linear Equation	R^2^	Linear Range	LOQ (mg/kg)
1	Butylated hydroxyl toluene	205	Y = 265423.6X + 8919.231	0.9997	0.5–10 mg/L	10
2	Octamethylcyclotetrasiloxane (D_4_)	281	Y = 395401.1X + 42389.18	0.9992	0.5–10 mg/L	10
3	Naphthalene	128	Y = 277563.0X − 4787.182	0.9996	0.5–10 mg/L	10
4	Dimethyl phthalate	163	Y = 97974.98X − 682.1819	0.9985	0.5–20 mg/L	10
5	Diethyl phthalate	149	Y = 103250.4X − 18509.75	0.9959	0.5–20 mg/L	10
6	Diisobutyl phthalate	223	Y = 10741.26X − 2167.55	0.9953	0.5–20 mg/L	10
7	Dibutyl phthalate	223	Y = 9305.55X − 2326.909	0.9957	0.5–20 mg/L	10
8	Benzyl butyl phthalate	206	Y = 16723.72X − 10804.66	0.9912	0.5–20 mg/L	10
9	Bis(2-ethyl hexyl) phthalate	279	Y = 11547.93X − 4729.022	0.9938	0.5–20 mg/L	10
10	Di-n-octyl phthalate	279	Y = 15814.77X − 9021.497	0.9931	0.5–20 mg/L	10
11	Diisononyl phthalate	293	Y = 14183.43X − 198.474	0.9975	0.5–20 mg/L	10
12	Diisodecyl phthalate	307	Y = 17146.42X − 3414.892	0.9980	0.5–20 mg/L	10
13	Isopropyl alcohol	45	Y = 62567.27X + 28998.92	0.9997	0.5–10 μg	10
14	1,4-dichlorobenzene	146	Y = 1446317X − 69461.15	0.9984	0.1–5 μg	10
15	Methanol	31	Y = 938.3207X − 3526.788	0.9979	5–100 mg/L	100
16	Benzaldehyde	-	Y = 0.13X + 0.16	0.9996	1–50 mg/L	10
17	Zinc oxide (analyzed as zinc)	-	Y = 3947829X + 5117.1	0.9999	0.05–10 mg/L	100

**Table 7:** Results of an analysis of 17 target ingredients from deodorizing agents.

Order	Chemicals	No. of Products (Detection Rate, %)	Concentration Range(mg/kg)			
			Max	Min	Sample ID	Max Conc.
1	Butylated hydroxyl toluene	30/47 (63.83)	154.605	18.517	D-S-I-1~7: 7/7	91.294
					D-S-A-1~11: 11/11	154.605
					D-S-C-1~7: 6/7	130.069
					D-S-S-1~4: 2/4	55.075
					D-L-I-1~5: 4/5	76.689
2	Zinc oxide	3/47 (6.38)	649	37	D-S-W-1~2: 1/2	649
					D-S-S-1~4: 2/4	95
3	Octamethyl cyclotetrasiloxane	4/47 (8.51)	218.314	11.412	D-S-I-1~7: 1/7	218.314
					D-S-A-1~11: 1/11	35.757
					D-S-W-1~2: 1/2	11.413
					D-L-I-1~5: 1/5	110.920
4	Isopropyl alcohol	2/47 (4.26)	236266	27	D-S-A-1~11: 1/11	236,266
					D-S-C-1~7: 2/7	27
5	Dibutyl phthalate	1/47 (2.13)	NA ^a^	NA	D-S-A-1~11: 1/11	358.860
6	Bis(2-ethyl hexyl) phthalate	1/47 (2.13)	NA	NA	D-S-A-1~11: 1/11	215.950
7	Di-n-octyl phthalate	3/47 (6.38)	100.690	24.152	D-S-I-1~7: 1/7	100.690
					D-S-C-1~7: 2/7	52.914
Other ingredients were not detected (<Limit of quantitation)						

^a^: NA: not available, because the subcategory consists of a single product sample.

**Table 8:** Results of a dermal risk assessment for butylated hydroxyl toluene.

Chemicals	Exposure Route	Detailed Risk Assessment Equation	RfD (mg/kg/day)	Application Type	Exposure Dose (mg/kg/day) (Max Conc.)	Target MOE (for Fabric/Shoes)		Calculated MOE
						Screening	Detail	
Butylated hydroxyl toluene	Dermal	D_der_ = R·t·W_f_·Abs·n/BW R: rate at which product is applied to the skin (mg/min)	25.0	Spray and trigger type	0.04895 (154.605 mg/kg)	1000	100	510

RfD: reference dose, MOE: margin of exposure.
